# Dynamic Modeling of the Multiring Disk Resonator Gyroscope

**DOI:** 10.3390/mi10030181

**Published:** 2019-03-10

**Authors:** Qingsong Li, Dingbang Xiao, Xin Zhou, Zhanqiang Hou, Ming Zhuo, Yi Xu, Xuezhong Wu

**Affiliations:** College of Intelligence Science, National University of Defense Technology, Changsha 410073, China; liqingsong336@163.com (Q.L.); zhouxin_schuman@yeah.net (X.Z.); houzhanqiang@nudt.edu.cn (Z.H.); zhuoming592@163.com (M.Z.); xuyi199206@163.com (Y.X.); xzwu@nudt.edu.cn (X.W.)

**Keywords:** mathematical modeling, dynamic model, disk resonator gyroscope, component mode synthesis method

## Abstract

The multiring disk resonator gyroscope (DRG) has been a candidate for high performance gyroscopes, however nowadays the finite element method (FEM) is the main method for its analysis due to its complex structure. In this paper we propose a new method to mathematically model the DRG for its vibrating modes and lumped parameters based on the component mode synthesis (CMS) method. Firstly, the natural frequencies and the associated mode shapes of the DRG are mathematically modeled and a comparison with the FEM results is conducted. It shows that the mode shapes of DRG obtained by FEM and mathematical modeling are identical and in the full ranges of geometrical parameters, natural frequency error of the simulation, and calculation results are limited in ±15%. It demonstrates the effectivity and feasibility of the mathematical modeling method. Then, based on the calculated natural frequencies and mode shapes, the lumped mass-spring model of the DRG and effects of geometry parameters on the lumped mass-spring parameters are investigated, which can be used on the design of the DRG. This mathematical modeling method can effectively improve the analyzing efficiency of the DRG and the method can also be used on the analysis of other complex multiring-type resonators.

## 1. Introduction

Gyroscopes are used to measure the rate of rotation (rate gyroscopes) or the angles of turn (angle gyroscopes), and various microgyroscopes have been reported in the literature [1,2,3]. Among them, the micromachined disk resonator gyroscope (DRG) working on the degenerate modes attracted much attention for its advantages of intrinsic mode matching, high quantity factor, high shock resistance, and less sensitivity to environment vibrations [4,5,6,7,8,9]. The degenerate modes include driving mode and sensing mode which ideally occur at the same frequency but have indistinguishable mode shapes. Generally, the in-plane fundamental (*n* = 2) flexural modes, also known as the wineglass mode, are utilized for rotating rate measurement in the DRG [10]. For the vibratory gyroscope in vibrating mode, the natural frequencies and the associated mode shapes are the most fundamental and important parameters. Calculation of the other performances of the gyroscope, such as effective mass, effective stiffness, mechanical sensitivity, quality factor, resolution, and others, are all based on the natural frequencies and the associated mode shapes [11,12].

In the previous literatures, natural frequencies and mode shapes are usually obtained by finite elements analysis (FEA) with the simulating software [13,14]. However, for a complex structure, it will take a long time to do the simulation and the meshing density has a great influence on the accuracy of simulating results. Meanwhile, with the FEA method, only the numerical solution can be obtained which cannot reflect the inner relationship between the results and the parameters. Therefore the development of a novel method for mathematical modeling of the frequencies and the associate mode shapes of a vibratory gyroscope is in urgent.

So far, many literatures have presented mathematical modeling of the wineglass mode of vibratory gyroscopes with simple structures. Refs. [15,16,17] presented the in-plane free vibration of a single-crystal silicon ring, Refs. [18,19] presented the detailed model of the semicircular springs supported ring gyroscope, and Refs. [11] presented the mathematical modeling of three-dimensional wineglass mode gyroscope. However, as for the complex multiring DRGs, no method has been proposed for the mathematical modeling.

In the vibrating DRG, modes of the different rings are different, meanwhile, the modes of each ring are not standard fundamental (*n* = 2) flexural modes. Thus, it is difficult to obtain the mathematical model of DRGs by utilizing the traditional method. In this paper, a method based on the component mode synthesis (CMS) [20,21,22,23,24,25,26] technique is proposed to mathematically model the complex DRGs. In the following sections, the design model and working principle of DRGs will be firstly described and then the CMS technique is introduced. Then, the calculation method based on CMS is used to calculate the natural frequencies and mode shapes of both the free vibrating and central-supported DRGs. Then, results of the mathematical modeling and FEA method are compared to verify the feasibility of the mathematical analysis method. Last, the calculated natural frequencies and mode shapes are used for calculation of the lumped mass-spring model of the DRG and the effects of geometry parameters on the lumped mass-spring parameters are investigated.

## 2. Materials and Methods

### 2.1. Design Model and Working Principle of the DRG

**Figure 1:**
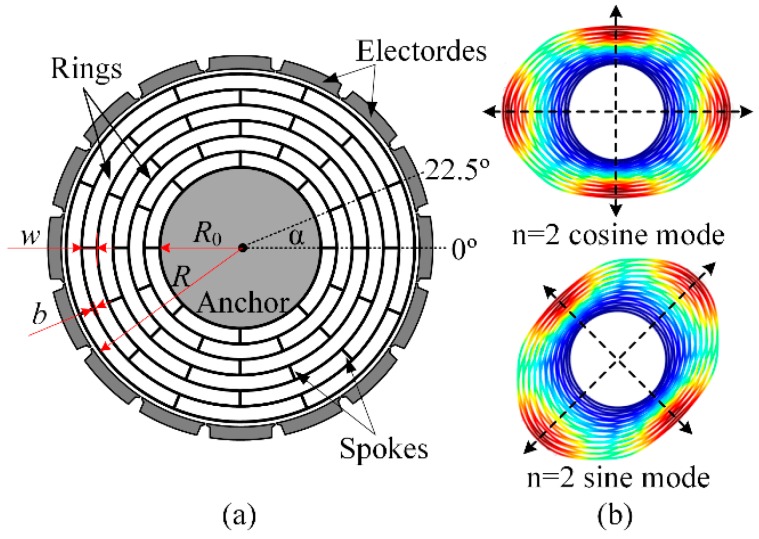
(**a**) Design model and (**b**) degenerate working modes of the typical disk resonator gyroscope.

The design model of a typical DRG is shown in Figure 1a [5,6,7,8,9,10,13,14,27,28,29]. The DRG consists of multiple concentric rings which are interconnected through 8 spokes on one layer that are anchored at the center. Sixteen electrodes are set around the sensing structure for driving, sensing and tuning. The DRG has a degenerate pair of in-plane wineglass modes, also called *n* = 2 cosine and sine modes which have the identical natural frequency but indistinguishable mode shapes, as shown in Figure 1b. It is driven to vibration at one of the wineglass modes and the Coriolis-based rotation rate signal is detected at another wineglass mode. As the *n* = 2 sine mode can be easily obtained by transferring the vibrating orientation of cosine mode from 0° to 45°, in this paper, only the cosine mode is analyzed to simplify the analyzing process.

### 2.2. Component Mode Synthesis Technique

CMS techniques are widely used for the dynamic analysis of complex structures [20,21,22,23,24,25,26,30,31]. They can be classified as fixed-interface methods, free-interface methods, and hybrid methods, depending upon whether the mode shapes used to define substructure coordinates are obtained with the interface coordinates fixed, free, or a combination of them. Normally, the fixed-interface methods can produce a better accuracy; however it is more complex than the free-interface methods. So in this paper, the free-interface method is used.

Firstly, the entire system needs to be partitioned into multiple individual substructures and the substructures are modeled individually. Secondly, dynamic models of all the substructures are simply assembled to produce an uncoupled model of the entire structure. Thirdly, the coupling of interfaces is built and the assembled uncoupled model is transferred to the coupled model of the entire system. At last, by solving the coupled model, the natural frequencies, mode shapes, and dynamic equation can be obtained. In the following section, mathematical modeling of the DRG is conducted following the above steps.

### 2.3. Mathematical Modeling of Free Vibrating DRGs

#### 2.3.1. Substructures Partition

**Figure 2:**
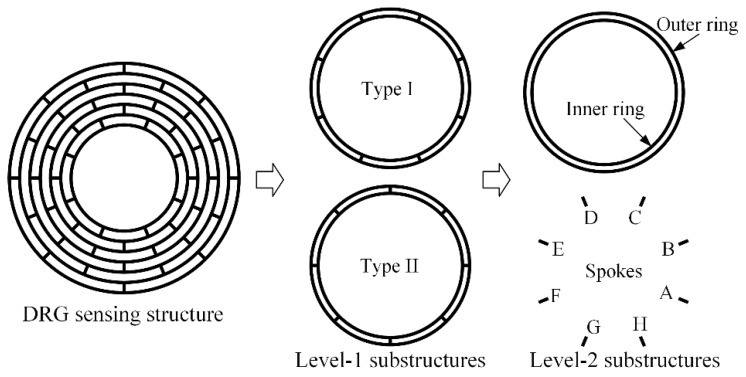
Substructures partition of the DRG sensing structure.

Based on the periodic characteristic of the DRG sensing structure, the adjacent two rings and the interconnected 8 spokes can be partitioned to the first level (level-1) substructures, while the single ring and spoke can be seen as the second level (level-2) substructures, as shown in Figure 2. While there are n-1 level-1 substructures in a DRG with n rings, there are 10 level-2 substructures (inner rings, outer rings, and spoke A-H) in a level-1 substructure. As the spokes of adjacent layers are alternately arranged, the level-1 substructures can be divided into two types: type I, in which spoke A is located at −22.5°, and type II, in which spoke A is located at 0°.

#### 2.3.2. Mathematical Modeling of the Level-2 Substructures

The study of the free vibrations of beams, curved bars, and rings can be traced back to the nineteenth century. Many hypothesis, theories, and methods have been proposed to solve the problem, such as the Timoshenko beam theory, the Euler–Bernoulli hypothesis, the Love hypothesis, the Hamilton’s principle, the Ritz method, among others [16,17,32,33,34]. In this paper the Ritz method is used for the mathematical modeling of the level-2 substructures including rings and spokes.

**Figure 3:**
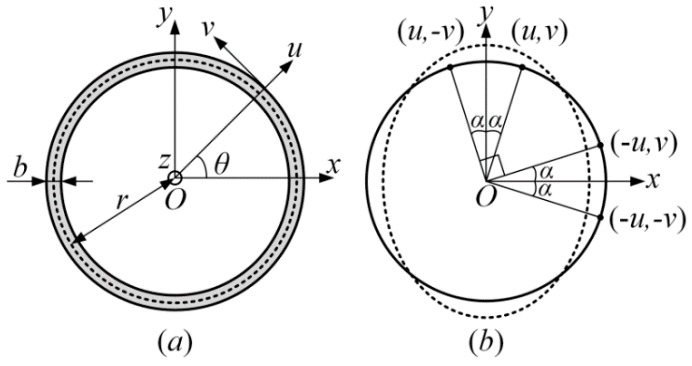
(**a**) Schematic of the single ring and (**b**) vibrating characteristic of its *n* = 2 cosine mode.

Consider a ring, as shown in Figure 3a, of radius *r*, width *b*, and thickness *h*, respectively. *θ* is the angle measured anticlockwise from the *x* axis. *u* and *v* are the radial and tangential displacements of the point on the centerline in polar coordinates. Normally, the width of the ring is so small compared to the radius that it can be seen as a thin ring and we adopt the Euler–Bernoulli hypothesis (that is, the plane of the cross-section remains plane which is perpendicular to the longitudinal axis during deformation and there is no Poisson’s effect) for the analysis of the ring [16]. As proposed in the literature [18,19], the vibrating ring can be analyzed using normal modes. To this end, any vibration-induced displacements of the ring can be expressed as the superposition of a series of normal vibration modes:

(1) $$\begin{array}{l} v(\theta ,t)=\sum_{N=1}^{\infty }\left[p_{N}^{r}(t)\sin N\theta +q_{N}^{r}(t)\cos N\theta \right]\\ u(\theta ,t)=\sum_{N=1}^{\infty }\left[p_{N}^{r}(t)N\cos N\theta -q_{N}^{r}(t)N\sin N\theta \right] \end{array}$$

Herein, in the thin ring with small deformation, *u* = *v*′. Dot and prime denote the derivative with respect to time *t* and angle *θ*, respectively. $p_{N}^{r}(t)$ and $q_{N}^{r}(t)$ are the generalized (modal) coordinates while cos*Nθ* and sin*Nθ* are the single ring’s normal modes. In this paper, only the *n* = 2 cosine mode of the DRG is analyzed to simplify the analyzing process. Since the cosine mode is symmetrical about the x axis at any time,

(2) u(0+α)=u(0−α)

where α is random angle. Expanding Equation (2) with Equation (1), and simplifying the equation, we get

(3) $$\sum_{N=1}^{\infty }\left[2q_{N}(t)\sin N\alpha \right]=0$$

This function always exists with random angle α, requiring that the generalized coordinates

(4) $$q_{N}^{r}(t)=0$$

This means that in the cosine mode of the DRG, the vibration mode of any ring only consists of cosine modes while sine modes do not exist. So the vibration mode of any ring can be expressed as

(5) $$\begin{array}{cc} v(\theta ,t)=\sum_{N=1}^{\infty }\left(p_{N}^{r}(t)\sin N\theta \right), & u(\theta ,t)=\sum_{N=1}^{\infty }\left(p_{N}^{r}(t)N\cos N\theta \right) \end{array}$$

Theoretically, the modes of each ring need to be expressed using the superposition of infinite normal modes, but actually, the high-order modes have smaller proportion than the low-order modes. More mode components will result in higher accuracy but also lead to a larger calculation amount. So, based on the accuracy we prefer to achieve, some high order modes can be neglected. Meanwhile, the *n* = 2 cosine mode has two basic properties—symmetry and periodicity—as shown in Figure 3b. Firstly, the mode shape is symmetrical about the y axis, which means that

(6) $$u(\frac{\pi }{2}+\alpha )=u(\frac{\pi }{2}-\alpha )$$

Therefore we can derive that

(7) $$\sum_{j=1}^{\infty }p_{j}^{r}(t)\sin j\frac{\pi }{2}\sin j\alpha =0$$

This function always exists with random angle *α*. As *p_j_*(*t*) cannot all be zero there must be

(8) $$\sin j\frac{\pi }{2}=0$$

Which requires that *j* must be an even number, which is

(9) j=2,4,6,8⋯2N⋯

Another property of the mode shape is the periodicity. In the cos2*θ* mode, displacement will be reversed after 90 degrees, which means that

(10) $$u(\frac{\pi }{2}+\alpha )=-u(\alpha )$$

Then we can derive that

(11) $$(\cos j\frac{\pi }{2}+1)\cos j\alpha =0$$

which exists with random angle α. Therefore,

(12) $$\cos j\frac{\pi }{2}=-1$$

Which requires that *j* must be twice of the odd number, which is

(13) j=2,6,10,14,18⋯4N−2⋯

Besides, in this paper, the first *N_r_* orders of normal modes are selected as the mode components of each ring for the trade-off between the calculation amount and accuracy (mode number of ring *N_r_* = 3 in this paper). So displacements of each ring can be briefly expressed as

(14) $$\begin{array}{l} v=p_{1}^{r}\sin2\theta +p_{2}^{r}\sin6\theta +p_{3}^{r}\sin10\theta =\varphi (\theta )p^{r}(t)\\ u=p_{1}^{r}2\cos2\theta +p_{2}^{r}6\cos6\theta +p_{3}^{r}10\cos10\theta =\psi (\theta )p^{r}(t) \end{array}$$

Herein,

(15) $$\begin{array}{c} \varphi (\theta )=\left[\begin{array}{ccc} \sin2\theta & \sin6\theta & \sin10\theta \end{array}\right],\psi (\theta )=\left[\begin{array}{ccc} 2\cos2\theta & 6\cos6\theta & 10\cos10\theta \end{array}\right]\\ p^{r}(t)={\left[\begin{array}{ccc} p_{1}^{r}(t) & p_{2}^{r}(t) & p_{3}^{r}(t) \end{array}\right]}^{T} \end{array}$$

So the kinetic energy *T^r^* can be expressed as

(16) $$\begin{array}{ll} T^{r} & =\frac{1}{2}\int_{0}^{2\pi }\rho hbr\left({\left(\frac{\partial u}{\partial t}\right)}^{2}+{\left(\frac{\partial v}{\partial t}\right)}^{2}\right)d\theta =\frac{1}{2}\sum_{i=1}^{3}\sum_{j=1}^{3}{\dot{p}}_{i}^{r}{\dot{p}}_{i}^{r}\int_{0}^{2\pi }\rho hbr\left(\psi_{i}\psi_{j}+\varphi_{i}\varphi_{j}\right)d\theta \\ & =\frac{1}{2}\sum_{i=1}^{3}\sum_{j=1}^{3}m_{ij}^{r}{\dot{p}}_{i}^{r}{\dot{p}}_{j}^{r}=\frac{1}{2}{\dot{p}}^{r}{}^{T}m^{r}{\dot{p}}^{r} \end{array}$$

where, *ρ* is density of the material and mass matrix **m^r^** can be expressed as

(17) $$m^{r}=\pi \rho hbr\left[\begin{array}{ccc} 2 & 0 & 0\\ 0 & 5 & 0\\ 0 & 0 & 10 \end{array}\right]=\pi \rho hbrm_{0}^{r}$$

Similarly, the potential energy *U^r^* can be calculated by

(18) $$\begin{array}{ll} U^{r} & =\frac{1}{2}{\int_{0}^{2\pi }\frac{Ehb^{3}}{12r^{3}}\left(u+\frac{\partial^{2}u}{\partial^{2}\theta }\right)}^{2}d\theta =\frac{1}{2}\sum_{i=1}^{3}\sum_{j=1}^{3}p_{i}^{r}p_{j}^{r}\int_{0}^{2\pi }\frac{Ehb^{3}}{12r^{3}}\left(\psi_{i}+\psi_{i}^{\prime \prime }\right)\left(\psi_{j}+\psi_{j}^{\prime \prime }\right)d\theta \\ & =\frac{1}{2}\sum_{i=1}^{3}\sum_{j=1}^{3}k_{ij}^{r}p_{i}^{r}p_{j}^{r}=\frac{1}{2}p^{r}{}^{T}k^{r}p^{r} \end{array}$$

where, *E* is the Young’s modulus of the material; the stiffness matrix **k** is also a diagonal matrix which can be expressed as

(19) $$k^{r}=\pi \frac{Ehb^{3}}{12r^{3}}\left[\begin{array}{ccc} 0 & 0 & 0\\ 0 & 36 & 0\\ 0 & 0 & 576 \end{array}\right]=\pi \frac{Ehb^{3}}{12r^{3}}k_{0}^{r}$$

Take Equations (16) and (18) into the Lagrange’s equation of the free vibrating system as follows

(20) $$\frac{d}{dt}\left(\frac{\partial T^{r}}{\partial {\dot{p}}_{i}^{r}}\right)-\frac{\partial T^{r}}{\partial p_{i}^{r}}+\frac{\partial U^{r}}{\partial p_{i}^{r}}=0\left(i=1,2,3\right)$$

Then dynamic equation of the single ring can be obtained:

(21) $$m^{r}{\ddot{p}}^{r}+k^{r}p^{r}=0$$

**Figure 4:**
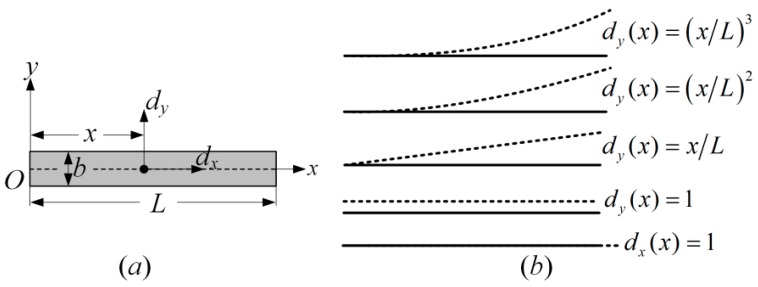
(**a**) Schematic of the single spoke and (**b**) its first five normal modes.

A schematic of the single spoke is shown in Figure 4a, where *b* and *L* are the width and length of the spoke, respectively. As for the free vibration of the Euler–Bernoulli beam structure, the x-axis displacement mainly includes the rigid displacement while neglecting the elastic displacement. The y-axis displacement can be expressed as the superposition of the rigid mode, rotating mode, and a series of normal flexural vibration modes. It can be expressed as

(22) $$\begin{array}{l} d_{x}(x)=p_{1}^{s}(t)\cdot 1\\ d_{y}(x)=p_{2}^{s}(t)\cdot 1+p_{3}^{s}(t)\cdot \frac{x}{L}+{\sum_{i=4}^{\infty }p_{i}^{s}(t)\left(\frac{x}{L}\right)}^{i-2} \end{array}$$

Similar to the ring structure, more mode components will result in higher accuracy but also lead to larger calculation amount. So, based on the accuracy we prefer to achieve, some high order modes can be neglected. In this paper, the first Ns order mode components are used (mode number of spoke *N_s_* = 5 in this paper). By simplifying the above equation, it can be expressed with the matrix form

(23) dx(x)=χ(x)ps(t)=[10000]ps(t)dy(x)=δ(x)ps(t)=[01xL(xL)2(xL)3]ps(t)

Herein,

(24) $$p^{s}(t)={\left[\begin{array}{ccccc} p_{1}^{s}(t) & p_{2}^{s}(t) & p_{3}^{s}(t) & p_{4}^{s}(t) & p_{5}^{s}(t) \end{array}\right]}^{T}$$

So the kinetic energy *T^s^* can be expressed as

(25) $$\begin{array}{ll} T^{s} & =\frac{1}{2}\int_{0}^{L}\rho hb\left({\left(\frac{\partial d_{x}}{\partial x}\right)}^{2}+{\left(\frac{\partial d_{y}}{\partial x}\right)}^{2}\right)dx=\frac{1}{2}\sum_{i=1}^{5}\sum_{j=1}^{5}{\dot{p}}_{i}^{s}{\dot{p}}_{j}^{s}\int_{0}^{L}\rho hb\left(\delta_{i}\delta_{j}+\chi_{i}\chi_{j}\right)d\theta \\ & =\frac{1}{2}\sum_{i=1}^{5}\sum_{j=1}^{5}m_{ij}^{s}{\dot{p}}_{i}^{s}{\dot{p}}_{j}^{s}=\frac{1}{2}{\dot{p}}^{s}{}^{T}m^{s}{\dot{p}}^{s} \end{array}$$

Herein,

(26) $$m^{s}=\rho hbL\left[\begin{array}{ccccc} 1 & 0 & 0 & 0 & 0\\ 0 & 1 & 1/2 & 1/3 & 1/4\\ 0 & 1/2 & 1/3 & 1/4 & 1/5\\ 0 & 1/3 & 1/4 & 1/5 & 1/6\\ 0 & 1/4 & 1/5 & 1/6 & 1/7 \end{array}\right]=\rho hbLm_{0}^{s}$$

Similarly, the potential energy *U^s^* can be expressed as

(27) $$\begin{array}{ll} U^{s} & =\frac{1}{2}{\int_{0}^{L}\frac{Ehb^{3}}{12}\left(\frac{\partial^{2}d_{y}}{\partial^{2}x}\right)}^{2}dx=\frac{1}{2}\sum_{i=1}^{5}\sum_{j=1}^{5}p_{i}^{s}p_{j}^{s}\int_{0}^{L}\frac{Ehb^{3}}{12}\delta_{i}^{\prime \prime }\delta_{j}^{\prime \prime }dx\\ & =\frac{1}{2}\sum_{i=1}^{5}\sum_{j=1}^{5}k_{ij}^{s}p_{i}^{s}p_{j}^{s}=\frac{1}{2}p^{s}{}^{T}k^{s}p^{s} \end{array}$$

Herein,

(28) $$k^{s}=\frac{Ehb^{3}}{12L^{3}}\left[\begin{array}{ccccc} 0 & 0 & 0 & 0 & 0\\ 0 & 0 & 0 & 0 & 0\\ 0 & 0 & 0 & 0 & 0\\ 0 & 0 & 0 & 4 & 6\\ 0 & 0 & 0 & 6 & 12 \end{array}\right]=\frac{Ehb^{3}}{12L^{3}}k_{0}^{s}$$

Then dynamic equation of the single spoke can also be obtained:

(29) $$m^{s}{\ddot{p}}^{s}+k^{s}p^{s}=0$$

#### 2.3.3. Mathematical Modeling of the Level-1 Substructures

**Figure 5:**
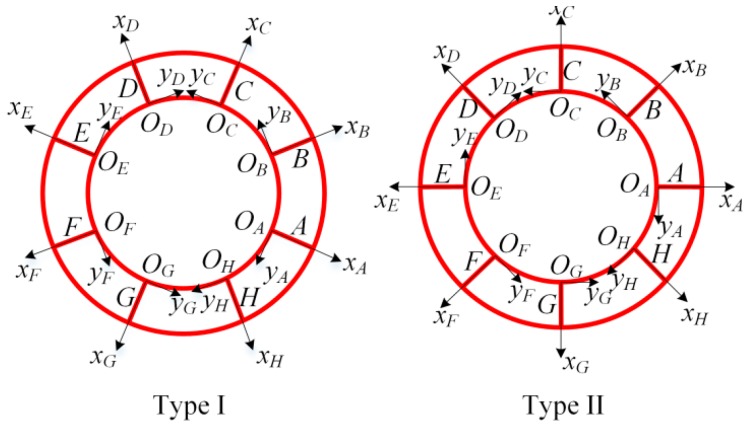
Schematic of the level-1 substructures.

Schematic of the level-1 substructures are shown in Figure 5. Based on the difference in the spokes’ orientations, they can be divided into two types: type I, in which the spoke A locates at −22.5°, and type II, in which the spoke A locates at 0°. In the *n* = 2 flexural mode of the DRG, the displacement will be reversed after 90 degrees, so spokes A and E have the same mode shape, while A, C, and G have reverse-phase mode shapes. Similarly, B and F have the same mode shape, while B, D, and H have reverse-phase mode shapes. So only the mode shapes of spokes A and B need to be analyzed. Uncoupled dynamic equation of the level-1 substructure *i* (including the ring *i*, ring *i* + 1 and the interconnected spokes) can be expressed as

(30) $$M_{0i}{\ddot{P}}_{i}+K_{0i}P_{i}=0$$

Herein, **M**_0*i*_, **K**_0*i*_, and **P***_i_* are the uncoupled mass matrix, stiffness matrix, and generalized coordinate of the level-1 substructure *i*, respectively. Therefore,

(31) $$\begin{array}{c} M_{0i}=diag\left(m_{i}^{r},m_{i+1}^{r},m_{iA}^{s},m_{iB}^{s}\right),K_{0i}=diag\left(k_{i}^{r},k_{i+1}^{r},k_{iA}^{s},k_{iB}^{s}\right)\\ P_{i}={\left[\begin{array}{cccc} p_{i}^{r} & p_{i+1}^{r} & p_{iA}^{s} & p_{iB}^{s} \end{array}\right]}^{T} \end{array}$$

**Figure 6:**
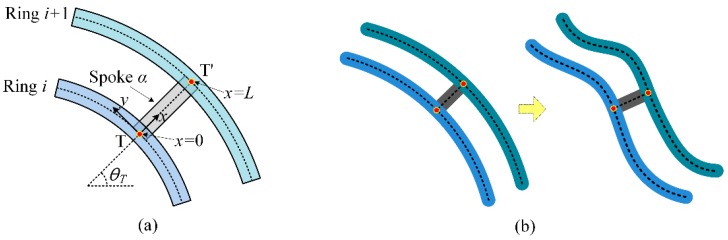
Schematic of (**a**) the joints and (**b**) the flexural deformation.

Joints of the rings and spokes are shown in Figure 6a. As the ring width is much smaller than the length of spoke and the radius of rings, it can be elucidated that the inner ring (ring *i*) and the spoke α (α = A, B, …, H) are connected together at the point T, which is also the cross point of the central line of the ring and spoke. Similarly, the spoke and the outer ring (ring *i* + 1) are connected at point T′. For the small flexural deformation as shown in Figure 6b, to ensure that the structure is still continuous, the rings and spokes must have the same radial displacement, tangential displacement, and rotating angle at points T and T′. So, the coupling condition on the interface of ring and spoke can be expressed as

(32) $$\begin{array}{cc} \begin{array}{l} u_{i}(\theta_{T})=d_{x}{}_{\alpha }(0),\\ v_{i}(\theta_{T})=d_{y\alpha }(0),\\ \phi_{i}(\theta_{T})=\sigma_{\alpha }(0), \end{array} & \begin{array}{l} u_{i+1}(\theta_{T})=d_{x}{}_{\alpha }(L)\\ v_{i+1}(\theta_{T})=d_{y\alpha }(L)\\ \phi_{i+1}(\theta_{T})=\sigma_{\alpha }(L) \end{array} \end{array}$$

As for a thin ring *i* at small deformation, the rotating angle *ϕ_i_* can be calculated as

(33) $$\phi_{i}=\frac{1}{r_{i}}\left(v_{i}+v_{i}{}^{\prime \prime }\right)=\frac{1}{r_{i}}\left(\psi (\theta_{T})+\psi^{\prime \prime }(\theta_{T})\right)p_{i}^{r}=\eta_{i}(\theta_{T})p_{i}^{r}$$

While, as for a thin beam *α* at small deformation, the rotating angle σ_α_ is

(34) $$\sigma_{\alpha }=\frac{\partial d_{y\alpha }}{\partial x}=\frac{\partial \delta (x)}{\partial x}p_{\alpha }^{s}=\varsigma (x)p_{\alpha }^{s}$$

By taking Equations (33) and (34) into Equation (32), the coupling of ring *i*, ring *i* + 1, and spokes A and spoke B can be obtained:

(35) ui(θT)=dxA(0),vi(θT)=dyA(0),ϕi(θT)=σA(0),ui+1(θT)=dxA(L)vi+1(θT)=dyA(L)ϕi+1(θT)=σA(L)ui(θT+π4)=dxB(0),vi(θT+π4)=dyB(0),ϕi(θT+π4)=σB(0),ui+1(θT+π4)=dxB(L)vi+1(θT+π4)=dyB(L)ϕi+1(θT+π4)=σB(L)

By taking the Equations (14), (15), (23), and (24) into Equation (35), the coupling relationships can be expressed with the following coupling matrix.

(36) [φ(θT)0−χ(0)0ψ(θT)0−δ(0)0ηi(θT)0−ς(0)00φ(θT)−χ(L)00ψ(θT)−δ(L)00ηi+1(θT)−ς(L)0φ(θT+π4)00−χ(0)ψ(θT+π4)00−δ(0)ηi(θT+π4)00−ς(0)0φ(θT+π4)0−χ(L)0ψ(θT+π4)0−δ(L)0ηi+1(θT+π4)0−ς(L)][pirpi+1rpiAspiBs]=0

Define the coupling matrix as **C***_i_*, it can be expressed as

(37) $$C_{i}\left(\theta_{T}\right)P_{i}(t)=0$$

As for the two types of level-1 substructures, *θ_T_* is different given that **C***_i_*(*θ_T_*) values for the two types of level-1 substructures are different. To simplify the analysis, we defined the innermost level-1 substructure as type I, as shown in Figure 2. Then, the coupling matrix **C***_i_*(*θ_T_*) of the level-1 substructure *i* is

(38) $$C_{i}=\{\begin{array}{cc} C_{i}(-\frac{\pi }{8}) & i=1,3,5,7\ldots \\ C_{i}(0) & i=2,4,6,8\ldots \end{array}$$

The mode number of each ring is *N_r_* (*N_r_* = 3) and the mode number of each spoke is *N_s_* (*N_s_* = 5), thus there are 12 rows and 2(*N_r_* + *N_s_*) columns in the coupling matrix and there are 2(*N_r_* + *N_s_*) generalized coordinates. However, due to the coupling between the rings and spokes, the number of the independent generalized coordinates is less than 2(*N_r_* + *N_s_*), which is the difference of the total mode numbers and rank of the coupling matrix *R*(**C***_i_*):

(39) $$N_{in}=2\left(N_{r}+N_{s}\right)-R(C_{i})$$

In this paper, *R*(**C***_i_*) is 11 and the number of independent generalized coordinates Nin is 5. By applying row elementary transformation on the coupling matrix **C***_i_*, the linearly independent coupling equation can be arranged at the first *R*(**C***_i_*) rows. Then, the coupling matrix **C***_i_* can be divided into four parts:

(40) $$C_{i}P_{i}=\left[\begin{array}{cc} C_{i}^{R(C_{i})\times N_{in}} & C_{i}^{R(C_{i})\times R(C_{i})}\\ 0 & 0 \end{array}\right]\left[\begin{array}{c} P_{i}^{N_{in}\times 1}\\ P_{i}^{R(C_{i})\times 1} \end{array}\right]=0$$

Herein, the vector of independent generalized coordinates, $P_{i}^{N_{in}\times 1}$, consists of all the generalized coordinates of ring *i* $P_{i}^{r}$ and part of the generalized coordinates of ring *i* + 1 ($P_{i}^{L}$). Defining the vector of independent generalized coordinates as **Q** we ascertain

(41) $$Q_{i}=P_{i}^{N_{in}\times 1}=\left[\begin{array}{c} P_{i}^{r}\\ P_{i}^{L} \end{array}\right]=\left[\begin{array}{c} P_{i}^{r}\\ p_{i+1\_1}^{r}\\ \vdots \\ p_{i+1\_N_{in}-N_{r}}^{r} \end{array}\right]$$

where, $p_{i+1\_x}^{r}$ is the x order generalized coordinate of the ring *i* + 1. Expanding (40) with (41) to obtain

(42) $$C_{i}^{R(C_{i})\times N_{in}}Q_{i}+C_{i}^{R(C_{i})\times R(C_{i})}P_{i}^{R(C_{i})\times 1}=0\Rightarrow P_{i}^{R(C_{i})\times 1}=-{\left[C_{i}^{R(C_{i})\times R(C_{i})}\right]}^{-1}C_{i}^{R(C_{i})\times N_{in}}$$

Then the relationship between all the generalized coordinates **P***_i_* and the independent generalized coordinates **Q***_i_* can be obtained:

(43) $$P_{i}=\left[\begin{array}{l} P_{i}^{N_{in}\times 1}\\ P_{i}^{R(C_{i})\times 1} \end{array}\right]=\left[\begin{array}{c} I^{N_{in}\times N_{in}}\\ -{\left[C_{i}^{R(C_{i})\times R(C_{i})}\right]}^{-1}C_{i}^{R(C_{i})\times N_{in}} \end{array}\right]Q_{i}=D_{i}Q_{i}$$

where, **D***_i_* is the transformation matrix of all the generalized coordinates **P***_i_* and of the independent generalized coordinates **Q***_i_*.

Take coupling Equation (43) into the uncoupled dynamic equation of the level-1 substructure (30), we can obtain the coupled dynamic equation of the level-1 substructure:

(44) $$M_{i}{\ddot{Q}}_{i}+K_{i}Q_{i}=0$$

Herein, **M***_i_* and **K***_i_* are the coupling mass matrix and stiffness matrix of the level-1 substructure *i*. Therefore,

(45) $$M_{i}=D_{i}{}^{T}M_{0i}D_{i},K_{i}=D_{i}{}^{T}K_{0i}D_{i}$$

#### 2.3.4. Mathematical Modeling of the Whole Sensing Structure

Similar to the mathematical modeling of the level-1 substructures, the CMS method needs to be used again for the mathematical modeling of the whole DRG structure.

**Figure 7:**
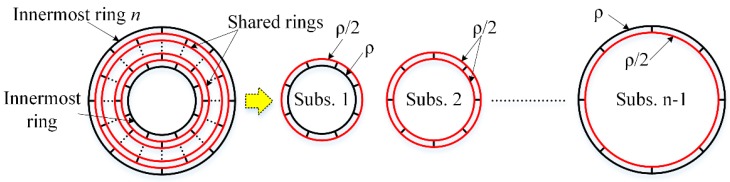
Schematic of the assembly of the level-1 substructures.

Level-1 substructures are assembled layer by layer to form the whole sensing structure, as shown in Figure 2. The adjacent level-1 substructures have a common ring, which is the outer ring of the inner level-1 substructure; also it is the inner ring of the outer level-1 substructure, as shown in Figure 7. So mass of the shared ring needs to be equally distributed to the adjacent two level-1 substructures, which can be achieved by defining the density of the shared rings as half of the original density of the material as shown in Figure 7. After the density of shared rings is changed, Equation (31) should also be modified as

(46) $$\begin{array}{l} M_{0i}=\{\begin{array}{ll} diag\left(m_{i}^{r},\frac{1}{2}m_{i+1}^{r},m_{A}^{s},m_{B}^{s}\right), & i=1\\ diag\left(\frac{1}{2}m_{i}^{r},\frac{1}{2}m_{i+1}^{r},m_{A}^{s},m_{B}^{s}\right), & i=2,3\ldots ,n-2\\ diag\left(\frac{1}{2}m_{i}^{r},m_{i+1}^{r},m_{A}^{s},m_{B}^{s}\right). & i=n-1 \end{array}\\ K_{0i}=\begin{array}{cc} diag\left(k_{i}^{r},k_{i+1}^{r},k_{A}^{s},k_{B}^{s}\right), & i=1,2,3\ldots ,n-1 \end{array} \end{array}$$

Then, given Equations (32)–(45), the mass matrixes Mi and stiffness matrixes **K***_i_* of each level-1 substructure can be calculated. Then the uncoupled model of the whole sensing structure can be expressed as

(47) $$M_{0}\ddot{Q}+K_{0}Q=0$$

Herein, **M_0_**, **K_0_**, and **Q** are the uncoupled mass matrix, stiffness matrix, and generalized coordinate of the whole sensing structure, respectively. Thus,

(48) $$\begin{array}{c} M_{0}=diag\left(M_{1},M_{2},\cdots ,M_{n-1}\right),K_{0}=diag\left(K_{1},K_{2},\cdots ,K_{n-1}\right)\\ Q={\left[\begin{array}{cccc} Q_{1} & Q_{2} & \cdots & Q_{n-1} \end{array}\right]}^{T} \end{array}$$

Equation (43) shows the relationship between all the generalized coordinates **P***_i_* and the independent generalized coordinates **Q***_i_* of the level-1 substructure *i*. Here, **D***_i_* can be divided into four parts according to the generalized coordinates of different level-2 substructures:

(49) $$P_{i}=\left[\begin{array}{c} p_{i}^{r}\\ p_{i+1}^{r}\\ p_{iA}^{s}\\ p_{iB}^{s} \end{array}\right]=\left[\begin{array}{c} D_{i}^{N_{r}\times \left(2N_{r}+2N_{s}\right)}\\ D_{i}^{N_{r}\times \left(2N_{r}+2N_{s}\right)}\\ D_{i}^{N_{s}\times \left(2N_{r}+2N_{s}\right)}\\ D_{i}^{N_{s}\times \left(2N_{r}+2N_{s}\right)} \end{array}\right]Q_{i}=\left[\begin{array}{c} D_{i}^{in}\\ D_{i}^{out}\\ D_{i}^{A}\\ D_{i}^{B} \end{array}\right]Q_{i}$$

Therefore,

(50) $$p_{i}^{r}=D_{i}^{in}Q_{i},p_{i+1}^{r}=D_{i}^{out}Q_{i}$$

As the adjacent level-1 substructures share the same ring as shown in Figure 7

(51) $$p_{i+1}^{r}=D_{i}^{out}Q_{i}=D_{i+1}^{in}Q_{i+1}$$

So the number of linearly independent coupling equations between the adjacent level-1 substructures is *N_r_* (*N_r_* = 3 in this paper). Then, once a level-1 substructure is added, number of the added independent generalized coordinates are *N_in_* ‒ *N_r_*. So the independent generalized coordinates of the whole sensing structure with *n* − 1 level-1 substructures can be expressed as

(52) $$\hat{Q}=\left[\begin{array}{ccccc} Q_{1} & p_{2}^{L} & p_{3}^{L} & \cdots & p_{n-1}^{L} \end{array}\right]$$

The relationship between the independent generalized coordinates of the adjacent level-1 substructures can be obtained as

(53) $$\begin{array}{ll} Q_{i+1} & =\left[\begin{array}{c} p_{i+1}^{r}\\ p_{i+1}^{L} \end{array}\right]=\left[\begin{array}{c} D_{i}^{out}Q_{i}\\ p_{i+1}^{L} \end{array}\right]=\left[\begin{array}{c} D_{i}^{out}\\ 0^{\left(N_{in}-N_{r}\right)\times N_{in}} \end{array}\right]Q_{i}+\left[\begin{array}{c} 0^{N_{r}\times \left(N_{in}-N_{r}\right)}\\ I^{\left(N_{in}-N_{r}\right)\times \left(N_{in}-N_{r}\right)} \end{array}\right]p_{i+1}^{L}\\ & =F_{i}Q_{i}+Gp_{i+1}^{L} \end{array}$$

So there is

$$\begin{array}{l} Q_{1}=IQ_{1}\\ Q_{2}=F_{1}Q_{1}+Gp_{2}^{L}\\ Q_{3}=F_{2}Q_{2}+Gp_{3}^{L}=F_{2}F_{1}Q_{1}+F_{2}Gp_{2}^{L}+Gp_{3}^{L}\\ ..................\\ Q_{n-1}=\prod_{i=n-2}^{1}F_{i}Q_{1}+\prod_{i=n-2}^{2}F_{i}Gp_{2}^{L}+\cdots +\prod_{i=n-2}^{m}F_{i}Gp_{m}^{L}+\cdots +Gp_{n-1}^{L} \end{array}$$

Therefore, the matrix form of the above equations is

(54) $$\left[\begin{array}{l} Q_{1}\\ Q_{2}\\ Q_{3}\\ \vdots \\ Q_{n-1} \end{array}\right]=\left[\begin{array}{lllll} I & 0 & 0 & \cdots & 0\\ F_{1} & G & 0 & \cdots & 0\\ F_{2}F_{1} & F_{2}G & G & \cdots & 0\\ \vdots & \vdots & \vdots & \vdots & \vdots \\ \prod_{i=n-2}^{1}F_{i} & \prod_{i=n-2}^{2}F_{i}G & \prod_{i=n-2}^{3}F_{i}G & \cdots & G \end{array}\right]\left[\begin{array}{l} Q_{1}\\ p_{2}^{L}\\ p_{3}^{L}\\ \vdots \\ p_{n-1}^{L} \end{array}\right]$$

Defining the coupling matrix between the level-1 substructures as S:

(55) $$Q=S\hat{Q}$$

Taking the coupling Equation (55) into the uncoupled dynamic equation of the whole sensing structure (47), we can get the coupled dynamic equation of the whole sensing structure:

(56) $$M\ddot{\hat{Q}}+K\hat{Q}=0$$

Herein, M and K are the coupled mass matrix and stiffness matrix of the whole sensing structure, respectively. Therefore,

(57) $$M=S^{T}M_{0}S,K=S^{T}K_{0}S$$

Generalized coordinate **Q** is the periodic function of the time t

(58) $$\hat{Q}=\xi \sin(\omega t+\varphi )$$

where, ω is the natural frequency, φ is the phase, and ξ is the undetermined coefficient vector. Taking (58) into (56):

(59) $$K\xi -\omega^{2}M\xi =0\Rightarrow M^{-1}K\xi =\omega^{2}\xi$$

Thus, the square of the angular natural frequencies is the eigenvalues of the dynamic matrix **M**^−1^**K**. Since we have simplified the mode components of each substructure according to vibration characteristics of the *n* = 2 flexural mode of the DRG, the minimum frequency is exactly the natural frequency ω_0_ of the preferred *n* = 2 cosine mode:

(60) $$\omega_{0}{}^{2}=\min(\lambda (M^{-1}K)),f_{0}=\frac{\omega_{0}}{2\pi }$$

Then, taking the value of ω_0_ into the equation:

(61) $$\left[K-\omega_{0}{}^{2}M\right]\xi_{0}=0$$

We can solve the corresponding eigenvector ξ_0_. The independent generalized coordinates can then be expressed as

(62) $${\hat{Q}}_{0}=\xi_{0}\sin(\omega_{0}t+\varphi )$$

By taking Equation (62) into Equations (54) and (55), the independent generalized coordinates of each level-1 substructure can be obtained:

(63) $$\left[\begin{array}{c} Q_{1}\\ Q_{2}\\ \vdots \\ Q_{n-1} \end{array}\right]=Q_{0}=S{\hat{Q}}_{0}=S\xi_{0}\sin(\omega_{0}t+\varphi )$$

The generalized coordinates of each level-2 substructure can also be obtained by Equation (49):

(64) $$\left[\begin{array}{l} p_{i}^{r}\\ p_{i+1}^{r}\\ p_{iA}^{s}\\ p_{iB}^{s} \end{array}\right]=\left[\begin{array}{l} D_{i}^{in}Q_{i}\\ D_{i}^{out}Q_{i}\\ D_{i}^{A}Q_{i}\\ D_{i}^{B}Q_{i} \end{array}\right],i=1,2,3\ldots n-1$$

Thus, the mode shape of all the rings and spokes in the DRG structure can be expressed as

(65) $$\begin{array}{ccc} \left[\begin{array}{c} u_{1}\\ u_{2}\\ u_{3}\\ \vdots \\ u_{n} \end{array}\right]=\left[\begin{array}{c} \psi (\theta )p_{1}^{r}\\ \psi (\theta )p_{2}^{r}\\ \psi (\theta )p_{3}^{r}\\ \vdots \\ \psi (\theta )p_{n}^{r} \end{array}\right], & \left[\begin{array}{c} v_{1}\\ v_{2}\\ v_{3}\\ \vdots \\ v_{n} \end{array}\right]=\left[\begin{array}{c} \varphi (\theta )p_{1}^{r}\\ \varphi (\theta )p_{2}^{r}\\ \varphi (\theta )p_{3}^{r}\\ \vdots \\ \varphi (\theta )p_{n}^{r} \end{array}\right], & \begin{array}{cc} \begin{array}{l} d_{x}{}_{A\_i}=\chi (x)p_{iA}^{s},\\ d_{x}{}_{B\_i}=\chi (x)p_{iB}^{s},\\ d_{y}{}_{A\_i}=\delta (x)p_{iA}^{s},\\ d_{y}{}_{B\_i}=\delta (x)p_{iB}^{s}. \end{array} & i=1,2\ldots n-1 \end{array} \end{array}$$

**Figure 8:**
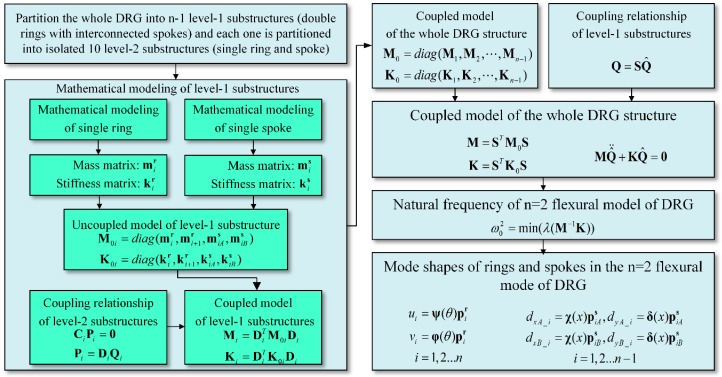
Analysis algorithm of the multiring DRG structure.

In summary, by using the analysis algorithm based on CMS technique, the natural frequency and mode shapes of each ring and spoke in the complex DRG structure can be calculated. Even though the simple analytical solution cannot be obtained due to the complex structure, the analysis algorithm can be easily achieved by computing software such as MATLAB and it is very useful for the analysis of the multiring resonators. The process of the algorithm is summarized in Figure 8.

### 2.4. Mathematical Modeling of the Central-supported DRG

In the above section, all the rings and spokes are assumed to vibrate freely. However, in disk resonator gyroscopes, the structure must be fixed to the substrate and the DRG is always central-supported to reduce the support damping, as shown in Figure 1. So in the multiring DRG model in Figure 2, the innermost ring should be completely fixed for the calculation. However, it is more complex for the mathematical modeling of a restrained structure than a free vibrating structure. So in this paper a simple way is proposed for the mathematical modeling of the central-supported DRG.

**Figure 9:**
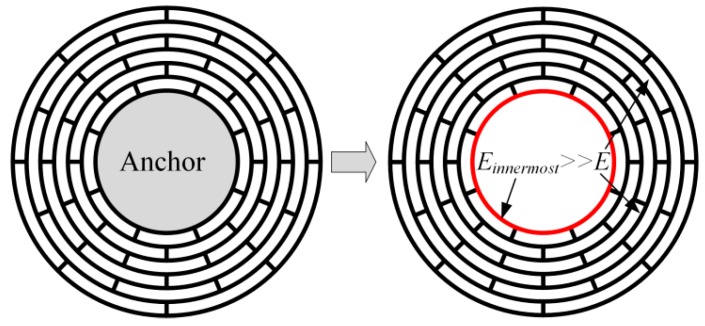
Effective model of the central-supported DRG by making the Young’s modulus of the innermost ring much larger than the other rings.

As known to us, the fixed ring has very small deformation in the vibration of the DRG, and it also can be achieved by greatly enlarging the stiffness of the innermost ring instead of fixing it. In this paper, the Young’s modulus of the innermost ring is set much larger than the other rings to achieve larger stiffness and small deformation, as shown in Figure 9. In this way, the analysis algorithms of the free vibrating DRG and the central-supported DRG are unified, which greatly decreases the complexity of the analysis algorithms of the central-supported DRG.

## 3. Results

### 3.1. Validation by Finite Element Analysis (FEA)

To verify the analysis algorithm for the natural frequencies and mode shapes of DRG proposed in this paper, FEA results are obtained with the commercial modeling tools software COMSOL, which is commonly used for this kind of simulation. The FEA results are compared with the calculation results obtained by MATLAB. The geometry parameters are shown in Figure 1. Thickness of the structure is set as 150 μm and the anchor radius is set as 1 mm. According to the common geometry parameters in micromachined DRGs, in the comparison, the range of the ring numbers, ring widths, and radius steps were set as 8–16, 2 μm–12 μm, and 40 μm–160 μm, respectively. To simplify the process, the ring widths and radius steps of different layers are set to be identical.

**Figure 10:**
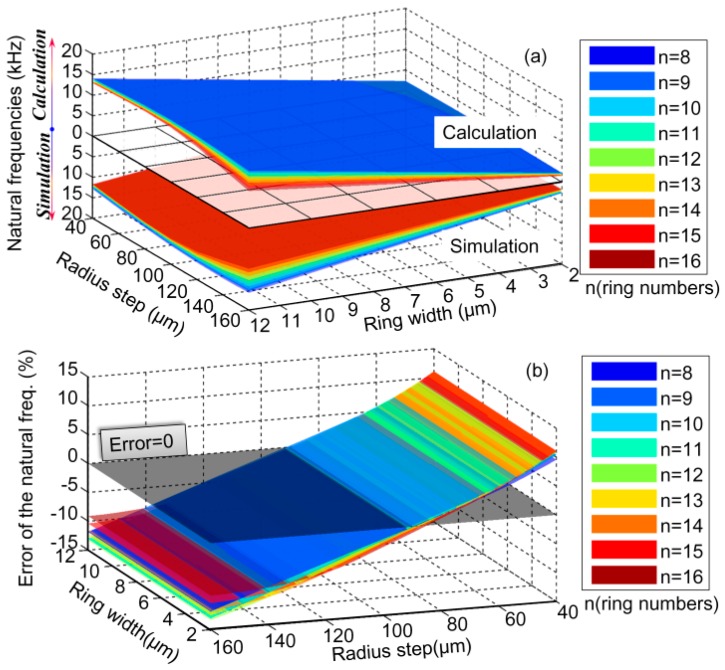
Comparison of natural frequencies of the free vibrating DRGs at different geometry parameters obtained by simulation and calculation: (**a**) the natural frequencies of the simulation and calculation results and (**b**) the relative error of the natural frequencies obtained by the two methods.

As for the free vibrating DRGs, the comparison of natural frequencies at different geometry parameters obtained by the simulation and calculation are shown in Figure 10a. Their relative errors at different geometry parameters are shown in Figure 10b. Here, the relative error of the simulation results (*f_s_*) and the calculation results (*f_c_*) is calculated by

(66) $$error=\frac{f_{s}-f_{c}}{f_{s}}\times 100\%$$

It can be seen that the changing rules of the simulated and calculated natural frequencies with the changing geometry parameters are identical. Meanwhile, in the full ranges of geometry parameters, the natural frequency error of the simulation and calculation results are limited to ±15%.

**Figure 11:**
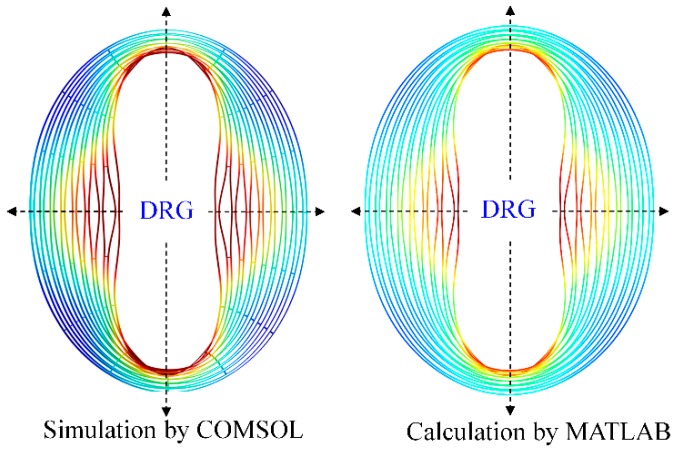
Comparison of the mode shapes of the free vibrating DRG which are simulated by COMSOL and calculated by MATLAB (spokes are neglected).

Mode shapes of the free vibrating DRG working at ~7.2 kHz (ring width, radius step, anchor radius and ring number are 6 μm, 36 μm, 1 mm, and 16, respectively) are obtained by simulation and calculation methods, as shown in Figure 11. It can be seen that the DRG mode shape obtained by simulation with the software COMSOL and the mode shape obtained by calculation with the software MATLAB are completely identical.

**Figure 12:**
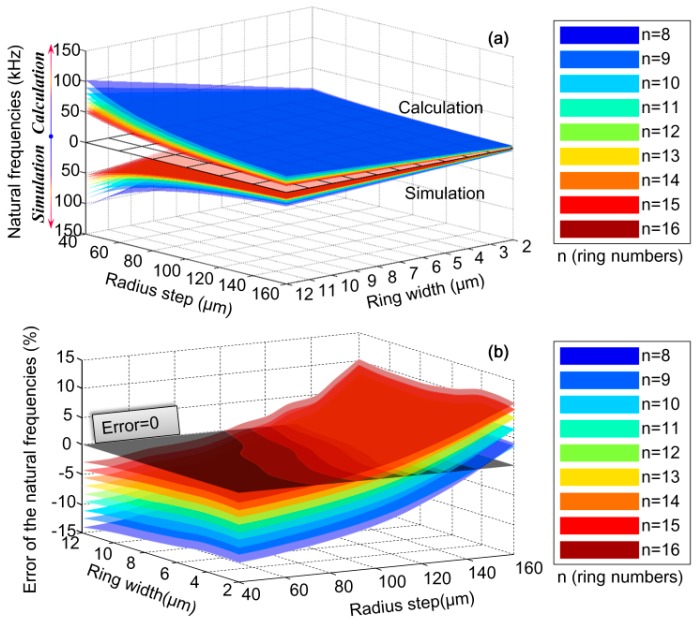
Comparison of natural frequencies of the central-supported DRG at different geometry parameters obtained by the simulation and calculation: (**a**) natural frequencies of the simulation and calculation results and (**b**) the relative error of the natural frequencies obtained by the two methods.

As for the central-supported DRGs, the comparison of the natural frequencies at different geometry parameters obtained by the simulation and calculation are shown in Figure 12. It can be seen that in the full ranges of geometry parameters the natural frequency error is also limited to ±15%. Errors come from both the simulation and calculation methods. The errors in simulation method mainly come from the meshing density of the structure. The errors in calculation method mainly come from the Euler–Bernoulli beam assumption, which is only suitable for the thin rings whose radius is much larger than the width.

**Figure 13:**
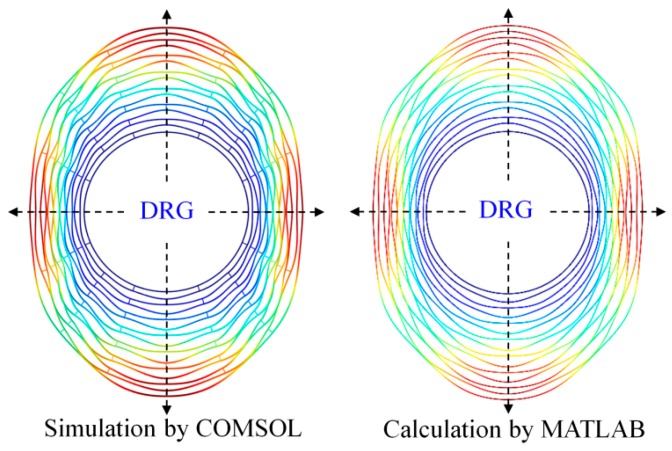
Comparison of the mode shapes of the central-supported DRG which are simulated by COMSOL and calculated by MATLAB (spokes are neglected).

Mode shapes of the central-supported DRG working at ~26 kHz (ring width, radius step, anchor radius and ring number are 10 μm, 70 μm, 1 mm, and 15, respectively) are shown in Figure 13. As is the same with the free vibrating DRG, the mode shapes obtained by the two methods are completely identical.

From Figure 10, Figure 11, Figure 12 and Figure 13, it can be learnt that the mathematical modeling algorithm for the analysis of the DRG natural frequencies and mode shapes proposed in this paper is effective and feasible. Although the FEA can also be used for the calculation of DRG natural frequencies, the mode shape function of every point in the structure cannot be obtained by this way. Furthermore, in order to get more accurate results, the meshing density of the FEA should be increased; however it will take a longer time for computation. For example, to get one group of frequency and mode shape of a DRG with certain structural parameters, it takes ~15 min with COMSOL; it only takes less than half a minute with MATLAB. To obtain the results in Figure 10a, we spend more than 7 days with COMSOL while only about 6 h is needed with MATLAB.

### 3.2. Derivation and Investigation of the Lumped Mass-Spring Model of DRG

The resonant frequency and mode shape obtained by the mathematical modeling can be applied on the investigation of the DRG lumped mass-spring model, the frequency splitting theory, the DRG performance analysis, and so on. Here, they are used for the investigation of the DRG lumped mass-spring model. In the analysis of gyroscopes, the lumped mass-spring model is always used instead of the distributed mass-spring model [3,11,12]. The mathematical model of the lumped model parameters is derived and the effects on them with different DRG geometry parameters are investigated.

The simplified motion equations of the DRG dynamic system can be expressed by [11,12]

(67) $$\begin{array}{l} m_{eff}{\ddot{q}}_{1}+m_{eff}\frac{\omega_{0}}{Q}{\dot{q}}_{1}+m_{eff}\omega_{0}{}^{2}q_{1}=F_{0}\sin(\omega_{0}t)\\ m_{eff}{\ddot{q}}_{2}+m_{eff}\frac{\omega_{0}}{Q}{\dot{q}}_{2}+m_{eff}\omega_{0}{}^{2}q_{2}=2\gamma \Omega_{z}\dot{x} \end{array}$$

where, *q*_1_ and *q*_2_ are the generalized displacement of the two normalized *n* = 2 flexural modes of DRG, which are separated by 45 degrees in Cartesian coordinates. *m_eff_* is the effective mass, *γ* is the Coriolis mass, *ω*_0_ is the angular resonant frequency, *Ω_z_* is the applied angular rate, and *F*_0_ is the amplitude of the driving force. *Q* is proportional to the inverse of total energy dissipated. Many dissipation mechanisms contribute to the total energy dissipation, such as thermoelastic dissipation (TED), anchor loss, air damping, etc. [35]. Therefore it is difficult to mathematically model the Q factor of DRGs, and this will be studied in our future work. In this study, the effects of the geometry parameters on the lumped mass-spring system parameters, including the effective mass, Coriolis mass, natural frequency, and angular gain, are investigated.

In the in-plane vibration, the vibration motion in the polar coordinate is expressed as the sum of the product of the generalized displacement (*q*_1_ and *q*_2_) and the corresponding normalized mode shape parameters (*ϕ_r_*_1_, *ϕ_θ_*_1_, *ϕ_r_*_2_, and *ϕ_θ_*_2_):

(68) $$U(\theta ,t)=\phi_{r1}(\theta )q_{1}(t)+\phi_{r2}(\theta )q_{2}(t),V(\theta ,t)=\phi_{\theta 1}(\theta )q_{1}(t)+\phi_{\theta 2}(\theta )q_{2}(t)$$

Herein, modes 1 and 2 denote the *n* = 2 cosine and sine mode of DRG:

(69) $$\left[\begin{array}{c} \phi_{r1}(\theta )\\ \phi_{\theta 1}(\theta ) \end{array}\right]=\frac{1}{q_{1}(t)}\left[\begin{array}{c} U_{1}(\theta ,t)\\ V_{1}(\theta ,t) \end{array}\right],\left[\begin{array}{c} \phi_{r2}(\theta )\\ \phi_{\theta 2}(\theta ) \end{array}\right]=\frac{1}{q_{2}(t)}\left[\begin{array}{c} U_{2}(\theta ,t)\\ V_{2}(\theta ,t) \end{array}\right]$$

In the mathematical modeling in Section 4, the generalized coordinates are corresponding to the normal modes of each ring and spoke. The motion equations and generalized coordinates corresponding to the normal modes are shown in Equations (56) and (65). So in order to get the lumped mass-spring model, the generalized coordinates corresponding to the normal modes need to be transferred to the generalized displacement corresponding to the normalized *n* = 2 flexural modes. By combining Equations (65) and (68), we can obtain the normalized *n* = 2 flexural modes of each ring:

(70) $$\phi_{r1\_i}(\theta )=\frac{u_{i}}{q_{1}}=\frac{1}{\max\left(\sqrt{u^{2}+v^{2}}\right)}\psi (\theta )p_{i}^{r},\phi_{\theta 1\_i}(\theta )=\frac{u_{i}}{q_{1}}=\frac{1}{\max\left(\sqrt{u^{2}+v^{2}}\right)}\varphi (\theta )p_{i}^{r}$$

where the contribution of spokes is neglected in the lumped mass-spring model, the *n* = 2 sine mode can be obtained by rotating the cosine mode for 45 degrees:

(71) $$\begin{array}{l} \psi_{2}(\theta )=\psi (\theta +\pi /4)=\left[\begin{array}{ccc} -2\sin2\theta & 6\sin6\theta & -10\sin10\theta \end{array}\right]\\ \varphi_{2}(\theta )=\varphi (\theta +\pi /4)=\left[\begin{array}{ccc} \cos2\theta & -\cos6\theta & \cos10\theta \end{array}\right] \end{array}$$

And we can obtain that

(72) $$\phi_{r2\_i}(\theta )=\frac{1}{\max\left(\sqrt{u^{2}+v^{2}}\right)}\psi_{2}(\theta )p_{i}^{r},\phi_{\theta 2\_i}(\theta )=\frac{1}{\max\left(\sqrt{u^{2}+v^{2}}\right)}\varphi_{2}(\theta )p_{i}^{r}$$

The maximum displacement in the cosine mode *q*_1_ can be replaced by the radial displacement amplitude of the point locates at 0 degree on the outermost ring. So the effective mass *m_eff_* can be calculated as [11,12]

(73) $$\begin{array}{ll} m_{eff} & =\sum_{i=1}^{n}\int_{0}^{2\pi }\rho hb_{i}r_{i}\left(\phi_{r1\_i}^{2}+\phi_{\theta 1\_i}^{2}\right)d\theta =\sum_{i=1}^{n}\int_{0}^{2\pi }\rho hb_{i}r_{i}\left(\phi_{r2\_i}^{2}+\phi_{\theta 2\_i}^{2}\right)d\theta \\ & =\frac{\pi \rho hb_{i}r_{i}}{{\left(\sum_{j=1}^{3}(j^{2}+1)p_{nj}\right)}^{2}}\sum_{i=1}^{n}\sum_{j=1}^{3}(j^{2}+1)p_{ij}^{2} \end{array}$$

The Coriolis mass γ can be calculated as

(74) $$\begin{array}{ll} \gamma & =\underset{V}{∭}\rho h\left(\phi_{r1}\phi_{\theta 2}-\phi_{r2}\phi_{\theta 1}\right)dV=\rho hb_{i}r_{i}\sum_{i=1}^{n}\sum_{j=1}^{3}\sum_{s=1}^{3}p_{ij}p_{is}\int_{0}^{2\pi }\left(\psi_{j}\varphi_{2}{}_{s}-\varphi_{j}\psi_{2}{}_{s}\right)d\theta \\ & =\frac{2\pi^{2}\rho hb_{i}r_{i}}{{\left(\sum_{j=1}^{3}(j^{2}+1)p_{nj}\right)}^{2}}\sum_{i=1}^{n}\sum_{j=1}^{3}\left[{\left(-1\right)}^{j+1}(4j-2)\right]p_{ij}^{2} \end{array}$$

The angular gain of DRG is defined as

(75) $$A_{g}=\frac{\gamma }{2M_{eff}}=\frac{\pi \sum_{i=1}^{n}\sum_{j=1}^{3}\left[{\left(-1\right)}^{j+1}(4j-2)\right]p_{ij}^{2}}{\sum_{i=1}^{n}\sum_{j=1}^{3}(j^{2}+1)p_{ij}^{2}}$$

**Figure 14:**
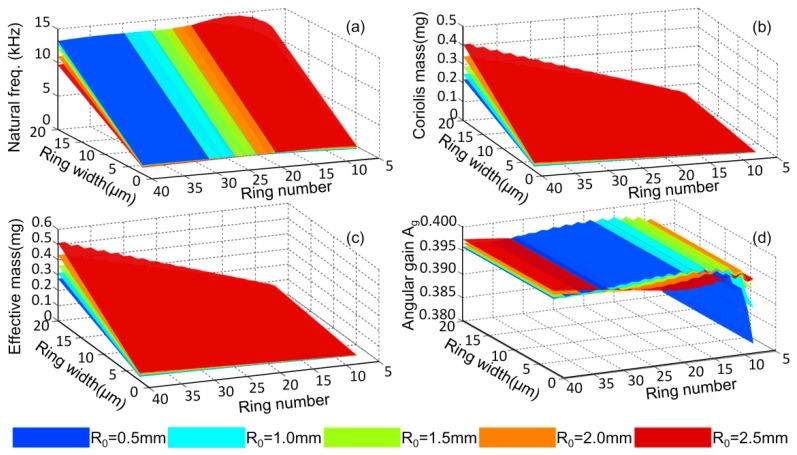
Effects of the geometrical parameters on (**a**) natural frequency, (**b**) Coriolis mass, (**c**) effective mass, and (**d**) angular gain of the DRG.

In the design of DRGs, the outer diameter of the DRG is always firstly confirmed based on the requirement of the sensor’s size and then the other parameters are confirmed based on the preferred performances. Here, the numerical solution and changing rules of the natural frequencies, effective mass, Coriolis mass, and angular gain with different geometrical parameters are studied as shown in Figure 14a–d. The outer diameter of the DRG is set at a constant 8 mm. The anchor radius, *R*_0_, ring number, *n*, and the ring width, *b*, ranging from 0.5 mm to 2.5 mm, 7 to 40, and 2 μm to 20, μm, respectively.

From Figure 14a, it can be seen that for a DRG with a certain diameter, the natural frequency is linear with relation to the ring width, while it has slight dependence on the other geometrical parameters. From Figure 14b,c, it can be learnt that the effective mass and Coriolis mass of the DRG are approximately linear with the ring width and the ring number. Figure 14d shows that the ring width has no influence on the angular gain of the DRG and the angular gain will be stable about 0.396 when the ring number is 10 or more. The changing rules can be used in the design of the DRG.

## 4. Conclusions

This paper proposes a method based on component mode synthesis (CMS) technique to mathematically model complex multiring DRGs for the first time. Firstly, the design model and working principle of multiring DRGs are described and then the CMS technique is used to calculate the natural frequencies and mode shapes of the DRGs. Comparison of natural frequencies and mode shapes obtained by the mathematical modeling and FEA methods demonstrates the feasibility and effectivity of the mathematical analysis method. At last, the natural frequencies and mode shapes are used for calculation of the lumped mass-spring model of the DRG and the effects of geometrical parameters on the lumped mass-spring parameters are investigated which is useful for the DRG design. As the mathematical model is theoretical in nature, it is unavoidable having differences with the experimental results of the fabricated devices. To make the theoretical results more accurate, this paper assumes that the geometrical parameters and material properties of the devices are identical with that used in the theoretical model; meanwhile, the devices are well fabricated without any defects and correctly tested. This mathematical modeling method can also be used on the analysis of other modes of the DRG or other multiring-type resonators.
